# Hematological and biochemical reference intervals of wild-caught and inhouse adult Indian rhesus macaques (*Macaca mulatta*)

**DOI:** 10.1186/s42826-022-00143-2

**Published:** 2022-11-11

**Authors:** Niraj A. Shah, Laxit K. Bhatt, Rajesh J. Patel, Tushar M. Patel, Nayankumar V. Patel, Harshida G. Trivedi, Nilam R. Patel, Jitendra H. Patel, Satish D. Patel, Rajesh S. Sundar, Mukul R. Jain

**Affiliations:** 1 Animal Research Facility, Zydus Research Centre, Zydus Lifesciences Limited, Ahmedabad, India; 2 Department of Pharmacology and Toxicology, Zydus Research Centre, Zydus Lifesciences Limited, Ahmedabad, India

**Keywords:** Safety evaluation, Domestic-born, Historical data, Reference range, Clinical pathology, Nonhuman primates

## Abstract

**Background:**

Nonhuman primates are used for research purposes such as studying diseases and drug discovery and development programs. Various clinical pathology parameters are used as biomarkers of disease conditions in biomedical research. Detailed reports of these parameters are not available for Indian-origin rhesus macaques. To meet the increasing need for information, we conducted this study on 121 adult Indian rhesus macaques (57 wild-sourced and 64 inhouse animals, aged 3–7 years). A total of 18 hematology and 18 biochemistry parameters were evaluated and reported in this study. Data from these parameters were statistically evaluated for significance amongst inhouse and wild-born animals and for differences amongst sexes. The reference range was calculated according to C28-A3 guidelines for reporting reference intervals of clinical laboratory parameters.

**Results:**

Source of the animals and sex appeared to have statistically significant effects on reference values and range. Wild-born animals reported higher WBC, platelets, neutrophils, RBC, hemoglobin, HCT, MCV, and total protein values in comparison to inhouse monkeys. Sex-based differences were observed for parameters such as RBCs, hemoglobin, HCT, creatinine, calcium, phosphorus, albumin, and total protein amongst others.

**Conclusions:**

Through this study, we have established a comprehensive data set of reference values and intervals for certain hematological and biochemical parameters which will help researchers in planning, conducting, and interpreting various aspects of biomedical research employing Indian-origin rhesus monkeys.

## Background

Non-human primates (NHPs) and humans share similarities in physiology and behavior. NHPs are an important animal model in the drug development process of pharmaceuticals and biologics. [1]. Various species of primates have been used in scientific studies including various apes, New World, and Old-World monkeys [2]. Out of all NHP species employed in research, macaques are the most widely used primates in basic and translational biomedical research. Macaques and humans share about 92% of genetic makeup as compared to the 64% similarity between humans and rodents, the latter being the most commonly used species in drug discovery and development [3]. Two species groups of the genus *Macaca*, the *M. fascicularis* or the cynomolgus monkeys and the *M. mulatta* or the rhesus monkey find extensive use in preclinical testing of various vaccines, monoclonal antibodies, and pharmaceuticals [3, 4].

The Indian-origin rhesus monkeys (*Macaca mulatta*) are a principal animal model for vaccine development and the study of HIV/AIDS pathogenesis [5]. They have been used as standard animal models for studying aging, neuroscience, and immunology. Benefits such as the discovery of the rhesus factor, development of certain life-saving vaccines like vaccines for rabies, polio, and smallpox, and understanding of embryonic stem cell propagation would not have been possible without the use of rhesus monkeys [5, 6]. Pharmaceutical and academic research centers in India rely heavily on the use of Indian-origin rhesus monkeys for toxicological studies as the primary nonrodent species of regulatory importance for the development of biologics. Due to their increased use in biomedical research, it is necessary to understand the effect of captivity on the hematological and biochemical parameters of the rhesus monkeys.

Historical reference values of clinical pathological parameters of Indian rhesus monkeys are scarcely available. Particularities such as methods of data collection, age, source of animals, methods of restraint, or environmental conditions are usually unclear or unavailable in most reports. Reference clinical pathology values are essential in the health monitoring of colonies, screening for healthy animals before employment in safety pharmacology and toxicology studies, and interpretation of laboratory data. Previous studies have reported variance in normal hematological and biochemical parameters due to differences in environmental conditions, confinement, and chemical restraint. Anaesthetization through ketamine hydrochloride is a common practice in the restraining of nonhuman primates. Published reports have described the effects of ketamine anesthesia on the hematological and biochemical parameters of rhesus monkeys [4]. In this study, clinical laboratory data has been obtained under a definite experimental set-up such as indoor housing, standard environmental and husbandry conditions, and acclimatized physical restraint procedures without anesthesia.

Despite their widespread use in biomedical research, few reports are available that compare and present differences in wild-caught and inhouse Indian rhesus monkeys. Studies have recommended that investigators consider the origin and history of the rhesus monkeys before they are evaluated for experimental purposes [6]. In the present study, we have aimed to prepare and present, accurate and statistically evaluated hematological and biochemical values of acclimatized and non-anesthetized individually-housed wild-caught and inhouse Indian rhesus monkeys.

## Results

### Reference values for hematological parameters

Different reference values for hematological parameters are reported in Table 1. Statistical analysis of reference values revealed varying degrees of statistical significance between inhouse and wild-sourced animals. Parameters such as WBC, RBC, HGB, HCT, MCH, MCHC, PLT, NEU, N%, L%, MONO, M%, EOS, and E% in males and WBC, HCT, MCV, MCH, MCHC, PLT, NEU, N%, L%. M%, E%, and EOS in females of wild-sourced animals were found to have a statistically significant difference in reference values when compared to inhouse animals. LYMP, BASO, and B% were the only comparable parameters among these animals. Markedly higher values of total leukocyte count along with increased NEU, N%, EOS, and platelet values and decreased L% and M% values were noticed in wild-sourced animals when compared to inhouse animals.

**Table 1:** Reference values for hematological parameters of Indian rhesus monkeys aged 3–7 years

Parameters (units)	Inhouse		Wild-sourced	
	Male (n = 42)	Female (n = 22)	Male (n = 42)	Female (n = 15)
WBC (10^3^/µL)	9.614 ± 2.870	9.820 ± 3.199	12.760 ± 3.496****	13.28 ± 4.486**
RBC (10^6^/µL)	5.691 ± 0.3545	5.525 ± 0.4232	5.904 ± 0.4568*	5.681 ± 0.5904
HGB (g/dL)	13.40 ± 1.705	12.98 ± 0.7787^$^	13.08 ± 0.9680*	12.47 ± 1.111^$^
HCT (%)	43.23 ± 3.601	41.17 ± 3.545^$^	45.60 ± 3.323**	44.58 ± 4.030*
MCV (fL)	75.95 ± 4.048	74.65 ± 4.676	77.35 ± 3.688	78.73 ± 5.020*
MCH (pg)	23.57 ± 2.804	23.57 ± 1.100	22.19 ± 1.189****	22.03 ± 1.614***
MCHC (g/dL)	31.07 ± 3.657	31.63 ± 1.555	28.69 ± 0.883****	27.99 ± 1.058****^$^
PLT (10^3^/µL)	360.7 ± 80.12	339.2 ± 84.69	548.0 ± 114.6****	548.9 ± 124.7****
NEU (10^3^/µL)	3.734 ± 2.060	4.653 ± 2.679	7.013 ± 2.962****	7.663 ± 3.533**
N% (%)	37.60 ± 13.28	43.44 ± 17.50	53.71 ± 10.99****	56.81 ± 14.46*
LYMP (10^3^/µL)	5.073 ± 1.467	4.474 ± 1.439	4.857 ± 1.366	4.685 ± 2.254
L% (%)	53.96 ± 12.10	47.83 ± 14.98	39.18 ± 9.704****	35.83 ± 12.54*
MONO (10^3^/µL)	0.5174 ± 0.3718	0.4359 ± 0.2982	0.3329 ± 0.1398**	0.4007 ± 0.1870
M% (%)	5.335 ± 3.123	6.235 ± 8.747	2.714 ± 1.156****	3.080 ± 0.9697*
EOS (10^3^/µL)	0.1864 ± 0.1303	0.1741 ± 0.1749	0.3688 ± 0.2928****	0.3280 ± 0.1086***
E% (%)	2.003 ± 1.368	1.809 ± 2.173	2.876 ± 1.894**	2.673 ± 1.227**
BASO (10^3^/µL)	0.06857 ± 0.05577	0.06045 ± 0.05376	0.05690 ± 0.02394	0.05467 ± 0.03583
B% (%)	0.6755 ± 0.5503	0.5395 ± 0.4590	0.4500 ± 0.1436	0.3933 ± 0.2120

Data are presented as mean ± SD, n = total number of samples. *p* < 0.05 is considered statistically significant

*= indicates varying degrees of statistical significance between inhouse and wild-sourced animals

^**$**^= indicates varying degrees of statistical significance between male and female animals.

The effect of sex on the hematological parameters of both inhouse and wild-sourced animals was minimal and limited to a few parameters. A statistically significant decrease was noted in the percent hematocrit of inhouse females and the mean corpuscular hemoglobin concentration of wild-sourced females when compared to inhouse and wild-sourced males respectively. Marginally decreased hemoglobin concentration in females of both inhouse and wild-sourced animals was observed when compared to respective male animals. Other parameters did not display any statistically significant difference between the sexes.

### Reference values for biochemical parameters

Different reference values for biochemical parameters are reported in Table 2. Similar to hematology, statistical analysis of biochemical parameters reference values revealed varying degrees of statistical significance between inhouse and wild-sourced animals. Parameters such as AST, ALT, GGT, TP, ALB, GLB, CREA, K^+^, and Ca^+^ in males and TCHO, GGT, TP, ALB, GLB, CREA, and K^+^ in females of wild-sourced animals were found to have a statistically significant difference in reference values when compared to inhouse animals. Important changes like a decrease in serum GGT, albumin, and creatinine and an increase in total protein, globulin, and potassium values were noted in wild-sourced animals when compared to inhouse animals. Another notable deviation found was a significant decrease in serum AST and calcium values of wild-sourced male animals when compared to inhouse males.

**Table 2:** Reference values for biochemical parameters of Indian rhesus monkeys aged 3–7 years

Parameters (units)	Inhouse		Wild-sourced	
	Male	Female	Male	Female
GLU (mg/dL)	79.18 ± 13.63 (42)	78.03 ± 15.30 (22)	87.01 ± 24.52 (42)	80.29 ± 22.87 (15)
TG (mg/dL)	62.18 ± 34.08 (41)	56.99 ± 21.08 (18)	53.23 ± 21.82 (42)	68.81 ± 29.92 (15)^$^
TCHO (mg/dL)	131.5 ± 28.00 (41)	131.8 ± 26.85 (18)	121.4 ± 22.04 (42)	112.6 ± 23.82 (15)*
AST (U/L)	46.98 ± 29.18 (42)	43.73 ± 15.37 (22)	36.45 ± 10.49 (42)*	41.83 ± 27.56 (15)
ALT (U/L)	54.56 ± 29.38 (42)	47.70 ± 20.84 (22)	37.92 ± 22.85 (42)****	37.91 ± 18.62 (15)
ALP (U/L)	490.5 ± 206.8 (41)	547.5 ± 245.1 (22)	548.9 ± 181.2 (42)	514.1 ± 157.6 (15)
GGT (U/L)	87.39 ± 60.03 (41)	74.88 ± 27.35 (17)	54.23 ± 10.18 (42)****	52.05 ± 10.59 (15)**
TBIL (mg/dL)	0.1855 ± 0.06653 (31)	0.1825 ± 0.05604 (16)	0.2079 ± 0.05010 (14)	0.2250 ± 0.06565 (6)
TP (g/dL)	7.943 ± 0.5061 (42)	7.618 ± 0.5270 (22)^$^	8.445 ± 0.5429 (42)****	8.320 ± 0.9420 (15)*
ALB (g/dL)	4.857 ± 0.4676 (42)	4.645 ± 0.4606 (22)^$^	4.455 ± 0.4717 (42)****	4.253 ± 0.6927 (15)*
GLB (g/dL)	3.086 ± 0.4409 (42)	2.973 ± 0.3561 (22)	3.990 ± 0.6374 (42)****	4.067 ± 1.109 (15)**
CREA (mg/dL)	1.016 ± 0.2007 (42)	0.8423 ± 0.1357 (22)^$$$$^	0.7367 ± 0.1336 (42)****	0.7287 ± 0.2359 (15)**
CK (U/L)	264.3 ± 295.6 (38)	291.6 ± 472.1 (22)	194.9 ± 102.0 (42)	197.6 ± 74.34 (15)
Na^+^ (mmol/L)	153.1 ± 3.524 (40)	153.2 ± 5.739 (17)	151.9 ± 3.944 (42)	151.3 ± 5.024 (15)
K^+^ (mmol/L)	4.742 ± 0.6323 (40)	4.385 ± 0.4047 (17)^$^	5.166 ± 0.7362 (42)**	5.157 ± 0.9364 (15)**
Cl^−^ (mmol/L)	105.9 ± 2.077 (40)	107.1 ± 3.650 (17)	105.5 ± 2.916 (42)	105.5 ± 2.646 (15)
Ca (mg/dL)	10.75 ± 0.5209 (42)	10.30 ± 0.3773 (22)^$$$^	10.41 ± 0.5331 (42)**	10.53 ± 0.7611 (15)
Phos (mg/dL)	5.988 ± 1.339 (42)	5.545 ± 1.404 (22)	6.343 ± 1.078 (42)	5.407 ± 1.220 (15)^$$^

Data are presented as mean ± SD

*p* < 0.05 is considered statistically significant. Numbers in parentheses indicate the sample size for individual parameters

* = indicates varying degrees of statistical significance between inhouse and wild-sourced animals, ^$^ = indicates varying degrees of statistical significance between male and female animals

The effect of sex was observed in a few biochemistry parameters in both inhouse and wild-sourced animals. A marked decrease was observed in serum creatinine and calcium levels of inhouse females when compared to inhouse males. Total protein and albumin were also found to have marginally lower values for inhouse females when compared to inhouse males. Wild-sourced female animals showed statistically significant changes such as an increase in serum triglycerides levels and a decrease in serum phosphorus levels when compared to wild-sourced male animals. Other remaining parameters displayed comparable results between sexes.

### Range of reference intervals

The range for reference intervals of hematological and biochemical parameters was calculated as per directions of the C28-A3 guideline. Reference intervals were compiled initially applying Grubbs’ test (alpha = 0.05) to identify outliers followed by estimations at 2.5th percentile (lower limit) and 97.5th percentile (upper limit). The calculated range of reference intervals with the number of samples for hematology and biochemistry parameters is reported in Tables 3 and 4 respectively.

**Table 3:** Range of reference intervals for hematological parameters of Indian rhesus monkeys aged 3–7 years

Parameters (units)	Inhouse		Wild-sourced	
	Male (n = 42)	Female (n = 22)	Male (n = 42)	Female (n = 15)
WBC (10^3^/µL)	4.42–14.38 (41)	5.34–12.66 (20)	5.91–19.62 (42)	4.49–22.07 (15)
RBC (10^6^/µL)	5.00–6.39 (42)	4.69–6.35 (22)	5.01–6.80 (42)	4.52–6.84 (15)
HGB (g/dL)	11.85–15.39 (41)	11.46–14.51 (22)	11.19–14.98 (42)	10.29–14.65 (15)
HCT (%)	36.17–50.29 (42)	34.22–48.12 (22)	39.08–52.11 (42)	39.51–51.12 (14)
MCV (fL)	68.02–83.88 (42)	65.49–83.81 (22)	70.12–84.58 (42)	68.89–88.57 (15)
MCH (pg)	21.91–26.04 (41)	21.41–25.72 (22)	20.26–24.30 (41)	20.28–24.42 (14)
MCHC (g/dL)	28.97–34.22 (41)	28.58–34.68 (22)	26.96–30.42 (42)	25.91–30.06 (15)
PLT (10^3^/µL)	203.64–517.69 (42)	173.19–505.18 (22)	323.37–772.59 (42)	355.32–694.54 (14)
NEU (10^3^/µL)	0.98–8.93 (42)	0.00–9.90 (22)	1.86–11.66 (41)	0.74–14.59 (15)
N% (%)	11.58–63.62 (42)	9.15–77.73 (22)	32.17–75.26 (42)	28.47–85.16 (15)
LYMP (10^3^/µL)	2.20–7.95 (42)	1.65–7.29 (22)	2.18–7.53 (42)	1.33–7.14 (14)
L% (%)	30.24–77.68 (42)	18.46–77.19 (22)	20.16–58.20 (42)	11.25–60.42 (15)
MONO (10^3^/µL)	0.13–1.12 (40)	0.04–1.15 (22)	0.09–0.55 (41)	0.14–0.58 (14)
M% (%)	1.83–11.70 (41)	0.00–8.92 (21)	1.30–5.70 (42)	1.18–4.98 (15)
EOS (10^3^/µL)	0.01–0.54 (42)	0.01–0.25 (20)	0.04–0.82 (40)	0.20–0.50 (14)
E% (%)	0.30–5.95 (42)	0.01–2.38 (20)	0.30–6.50 (41)	0.27–5.08 (15)
BASO (10^3^/µL)	0.01–0.16 (40)	0.01–0.20 (22)	0.02–0.12 (42)	0.00–0.10 (14)
B% (%)	0.00–2.33 (42)	0.00–1.35 (21)	0.20–0.80 (41)	0.00–0.81 (15)

Numbers in parentheses indicate the sample size employed in calculating the reference range after omission of outliers at 5% of significance, *n*, total number of animals

**Table 4:** Range of reference intervals for biochemical parameters of Indian rhesus monkeys aged 3–7 years

Parameters (units)	Inhouse		Wild-sourced	
	Male (n = 42)	Female (n = 22)	Male (n = 42)	Female (n = 15)
GLU (mg/dL)	52.46–105.90 (42)	48.05–108.02 (22)	38.94–135.08 (42)	35.47–125.11 (15)
TG (mg/dL)	21.00–136.10 (40)	15.67–98.32 (18)	18.00–108.10 (42)	25.77–99.57 (14)
TCHO (mg/dL)	76.58–186.34 (41)	79.15–184.40 (18)	78.19–164.59 (42)	65.90–159.26 (15)
AST (U/L)	9.51–74.07 (40)	19.70–63.10 (21)	17.68–53.57 (41)	20.16–49.77 (14)
ALT (U/L)	20.22–70.40 (37)	16.43–72.37 (21)	17.50–70.10 (41)	10.24–54.08 (13)
ALP (U/L)	51.95–905.71 (42)	67.19–1027.90 (22)	193.83–904.06 (42)	205.27–822.97 (15)
GGT (U/L)	32.67–120.74 (39)	21.27–128.49 (17)	34.27–74.19 (42)	31.29–72.81 (15)
TBIL (mg/dL)	0.03–0.30 (30)	0.07–0.29 (16)	0.11–0.31 (14)	0.10–0.35 (6)
TP (g/dL)	7.03–8.78 (41)	6.59–8.65 (22)	7.38–9.51 (42)	6.47–10.17 (15)
ALB (g/dL)	4.38–5.50 (40)	3.74–5.55 (22)	3.53–5.38 (42)	2.90–5.61 (15)
GLB (g/dL)	2.50–4.30 (42)	2.27–3.67 (22)	2.74–5.24 (42)	2.75–4.88 (14)
CREA (mg/dL)	0.62–1.41 (42)	0.69–0.97 (21)	0.47–1.00 (42)	0.27–1.19 (15)
CK (U/L)	57.46–321.59 (34)	18.42–366.95 (21)	85.00–386.00 (41)	78.95–289.20 (14)
Na^+^ (mmol/L)	145.00–159.00 (40)	141.99–164.48 (17)	144.18–159.63 (42)	141.49–161.18 (15)
K^+^ (mmol/L)	3.50–5.98 (40)	3.59–5.18 (17)	3.72–6.61 (42)	3.77–6.17 (14)
Cl^−^ (mmol/L)	101.82–109.97 (40)	99.90–114.21 (17)	99.77–111.20 (42)	100.36–110.73 (15)
Ca (mg/dL)	9.40–12.20 (42)	9.50–11.10 (17)	9.37–11.46 (42)	9.03–12.02 (15)
Phos (mg/dL)	3.36–8.61 (42)	3.17–7.55 (21)	4.23–8.46 (42)	3.02–7.80 (15)

Numbers in parentheses indicate the sample size employed in calculating the reference range after omission of outliers at 5% of significance, *n*, total number of animals

## Discussion

In the present study, we have reported standard reference values and ranges for various hematological and biochemical parameters of inhouse and wild-sourced adult (3–7 years old) Indian rhesus monkeys.

Previously, different studies have reported reference values for hematology and biochemistry parameters for rhesus monkeys [3, 4, 7, 8]. It is known that these reference values are affected by various parameters such as age, sex, fasting, sedation, or methods of restraint [3]. Some studies have either reported data of purpose-bred or wild-caught animals of different species [9, 10]. However, no reports are available that compare and analyze reference values for hematological and biochemical parameters in inhouse and wild-sourced Indian rhesus monkeys. Due to the increased use of rhesus monkeys in biomedical research, establishing thorough reference ranges of essential hematology and biochemistry parameters becomes essential in understanding health, biological variations, effects of drugs, and data interpretation. Hence, in this study, we have prepared, analyzed, and reported reference values and ranges for different hematological and biochemical parameters for adult (3–7 years of age) inhouse and wild-sourced Indian rhesus monkeys.

### Comparison between inhouse and wild-sourced animals

Stark differences were noticed in certain hematological parameters between inhouse and wild-sourced animals in the present study. Parameters such as total leukocyte counts, platelets, and certain differential leukocyte count parameters like absolute and percent neutrophils and eosinophils were noticed to be statistically higher in the wild-sourced animals than inhouse animals. An altered neutrophils-lymphocytes ratio was observed in wild-sourced animals. Neutrophils and lymphocytes form a large part of the total number of leukocytes in the blood [11]. It has been reported that sudden episodes of excitement or fright can provoke physiological leukocytosis in macaques, wherein, the leukocytes shift from the marginal pool to the circulating pool within a short time causing a marked increase in total leukocyte counts [11, 12]. This reaction is common for animals that are untrained and/or unanesthetized as the wild-caught animals in the present study. Higher levels of leukocytes are also a result of higher levels of circulating cortisol, a characteristic feature of capture-induced stress [12]. Lower or normalized levels of total leukocyte counts in inhouse animals can hence be attributed to increased adaptation to handling and captivity and succeedingly to, decreased stress and cortisol levels post handling or restraining. Additionally, notably higher absolute and percent leukocyte counts can be a result of exposure to various microbes in their natural environment. The increased leukocyte and platelet counts observed in the present study are similar to those observed in a study conducted on captive vervet monkeys [12]. The authors suggest that higher platelets in wild-caught animals in the present study could be attributed to capture-induced stress and subclinical infections. Acute mental stress has been observed to cause a significant increase in mental stress in humans [13].

Higher values were observed for RBC, Hemoglobin, and HCT parameters in this study compared to a different study conducted on the same species [14]. Wild-sourced male animals had the highest RBC counts, while inhouse male animals had the highest hemoglobin concentrations among all animals in the present study. Additionally, increased HCT and MCV and decreased MCH and MCHC values were found in both sexes of wild-sourced animals. These parameters remained comparatively stable in inhouse animals.

Few biochemistry parameters showed a marked difference from others in reference values of inhouse and wild-sourced animals. Liver health biomarkers such as AST, ALT, GGT, and albumin were statistically lower in wild-sourced than in inhouse animals. Serum creatinine and serum calcium to ascertain bone health were found to be in lower quantities in wild-sourced animals when compared to inhouse animals. Lower serum creatinine is indicative of good kidney function and hepatoprotection.

Slightly elevated levels of serum potassium were found in wild-sourced animals when compared to inhouse animals. Significantly increased total protein values were also noticed for wild-sourced animals. These significant differences in hematological and biochemical parameters of wild-sourced and inhouse animals can be attributed to several different factors such as type of food and feeding behavior of the animals, availability of food resources in the surroundings, physical attributes, inherent pathogenic infections, social status amongst large groups, environmental and living conditions in the wild and geographical location among others [6, 15–17].

Although marked differences exist between wild-sourced and inhouse animals, these animals could be used for experimental purposes if the hematology and biochemistry results fall under the normal reference range established for respective sources. Nonclinical safety evaluation studies warrant background data of animals before they are employed in specific studies. In such cases, animals with a mixed source of origins can be used for safety evaluation if source-specific historical data are available as presented in this study.

### Sex-based differences

Statistically significant differences amongst sexes were observed randomly in this study. Marginally lower hemoglobin was observed in inhouse and wild-caught females and lower HCT concentration was observed in inhouse females when compared to male animals of respective sourced animals. This difference can be owed to menstrual blood loss in females. Previously reported data from Matsuzawa 1993 showed marginally lower RBC counts in captive female rhesus monkeys, while hemoglobin concentration and hematocrit were comparable amongst sexes. Significant sex-based differences in erythrocyte count, hemoglobin concentration, and HCT have been reported for cynomolgus monkeys [18, 19], squirrel monkeys [9], and Chinese-origin rhesus macaques [20]. These findings can be correlated to gender-based differences in hematological parameters observed in humans [9, 21]. It is reported that lower RBC counts in female animals are a result of the inhibitory influence of estrogen on erythropoiesis. Statistically significant higher values of hemoglobin, HCT, and MCHC parameters in male animals in this study can be attributed to the production of male sex hormones and bigger muscle mass that require greater amounts of oxygen [12, 22].

Sex-based differences of statistical importance were observed for creatinine, calcium, and inorganic phosphorus parameters. Lower levels of serum calcium and creatinine in inhouse females and lower serum phosphorus levels in wild-sourced females were of significance when compared to male animals of respective sources. Other studies have previously reported minor differences in mean serum calcium and phosphorus levels amongst rhesus monkeys [9], while others have reported non-significance amongst sexes [20]. Similar to our study, a study conducted on Chinese-origin rhesus monkeys has also reported a statistically significant difference in serum total protein, albumin, and creatinine values when compared amongst sexes. Sex-based differences in parameters of wild-caught macaques could be a result of diet differences among males and females of varying societal hierarchies in the wild. Varying amounts of protein intake can additionally affect biological markers concentration in the blood. Inhouse animals are fed with standard monkey feed that is nutrient-balanced, and thus, fewer variations are observed in serum markers on inhouse animals.

## Conclusions

In conclusion, we have established baseline values of hematological and biochemical parameters with definite experimental conditions. The reference values presented in the present study might be representative of adult Indian rhesus monkeys housed under conditions identical to those in safety evaluation studies, and therefore, would serve as the basis for animal selection and safety/toxicology data interpretation. Further comprehensive evaluations are required that employ a large number of animals of varying age groups to prepare a thorough historical data range that will prove extremely useful in safety evaluation.

## Methods

### Animals

Results in the present study were obtained from 57 wild-sourced animals (42 males and 15 females) and 64 inhouse animals (42 males and 22 females) aged 3–7 years, housed at the Primate Research Facility of Zydus Lifesciences Limited at Zydus Research Centre in Ahmedabad, India. Wild-caught monkeys screened for the absence of *Mycobacterium tuberculosis* were obtained from a CPCSEA-known vendor under an official permit from the Ministry of Environment and Forest, Government of India (GoI). These animals were quarantined for a period of 6-weeks upon arrival at the Primate Research Facility, Zydus Research Centre, Ahmedabad. All animals were considered adults based on the age classification standards for humans to macaques – dental scale method [23]. Animals were housed individually in stainless-steel apartment-type cages with enrichment items. The environment was controlled to maintain a temperature of 18–29 °C, a 12:12 h light–dark cycle, and 15 air changes per hour. Animals were fed once daily with seasonal fruits and/or vegetables and a commercial NHP maintenance diet (6029-extrudate, Maintenance diet for non-human primates, Altromin Spezialfutter GmbH & Co. KG, Germany). Potable drinking water (reverse osmosis followed by UV treatment) was provided ad libitum. Facility veterinarians regularly examined all animals and monitored for the occurrence of diseases and changes in normal behavior.

### Blood sampling and analysis

Animals were fasted overnight. Blood was withdrawn from the cephalic or saphenous veins of non-anesthetized monkeys restrained in squeeze-back mechanism cages by trained personnel under the supervision of facility veterinarians. For hematology analysis, blood was collected in ready-to-use vacutainers containing K_2_-EDTA. Hematology parameters were analyzed using Advia 2120i hematology analyzer (Siemens Healthineers, USA). For serum biochemistry analysis, blood was collected in Gel + Clot activator tubes. Blood was allowed to clot for at least 30 min at room temperature before centrifugation at 4000 rpm for 10 min at 24 °C to obtain serum. Serum biochemistry parameters were analyzed using Cobas c311 analyzer (Roche Diagnostics, Switzerland). Blood samples were analyzed at Clinical Pathology Laboratory-II (CPL-II), Zydus Research Centre, Ahmedabad. Procedures followed were as per the applicable SOPs in place at the institution. The parameters evaluated, their abbreviations, units, and methods of analysis are presented in Table 5.

**Table 5:** Abbreviations, units of measurements and methods of analysis of different hematological and biochemical parameters

Parameters	Abbreviations	Units	Method of analysis
*Hematology*			
Total leukocyte count	WBC	10^3^/µL	Laser light scatter
Erythrocyte count	RBC	10^6^/µL	Light scattering—optical cytometer
Hemoglobin concentration	HGB	g/dL	Cyanide-free hemoglobin methods
Hematocrit	HCT	%	Calculated
Mean corpuscular volume	MCV	fL	Cumulative pulse height detection
Mean corpuscular hemoglobin	MCH	Pg	Calculated
Mean corpuscular hemoglobin concentration	MCHC	g/dl	Calculated
Platelet	PLT	10^3^/µL	Light scattering—optical cytometer
Neutrophil	NEU	10^3^/µL	Flow cytometry
Neutrophil percentage	N%	%	
Lymphocyte	LYMP	10^3^/µL	
Lymphocyte percentage	L%	%	
Monocyte	MONO	10^3^/µL	
Monocyte percentage	M%	%	
Eosinophil	EOS	10^3^/µL	
Eosinophil percentage	E%	%	
Basophil	BASO	10^3^/µL	
Basophil percentage	B%	%	
*Biochemistry*			
Glucose	GLU	mg/dL	Hexokinase method
Triglycerides	TG	mg/dL	Enzymatic colorimetric method
Total cholesterol	TCHO	mg/dL	Enzymatic colorimetric method
Aspartate aminotransferase	AST	U/L	IFCC method
Alanine aminotransferase	ALT	U/L	
Alkaline Phosphatase	ALP	U/L	
Gamma glutamyl transferase	GGT	U/L	Enzymatic colorimetric method
Total bilirubin	TBIL	mg/dL	Colorimetric diazo method
Total protein	TP	g/dL	Colorimetric biuret method
Albumin	ALB	g/dL	Bromocresol green method
Globulin	GLB	g/dL	Calculated
Creatinine	CREA	mg/dL	Jaffe method
Creatine kinase	CK	U/L	UV test
Sodium	Na^+^	mmol/L	ISE indirect
Potassium	K^+^	mmol/L	
Chloride	Cl^−^	mmol/L	
Calcium	Ca	mg/dL	NM-BAPTA method
Inorganic phosphorus	Phos	mg/dL	Phosphomolybdate UV method

*IFCC*, international federation of clinical chemistry, *ISE*, ion selective electrodes, NM-BAPTA: 5-nitro-5′-methyl-(1,2-bis(o-aminophenoxy)ethan-N,N,N′,N′-tetraacetic acid chromophore, *UV*, ultraviolet

### Ethics statement and accreditations

The animal facility is registered with The Committee for the Purpose of Control and Supervision of Experiments on Animals (CPCSEA), a statutory committee under the Ministry of Fisheries, Animal Husbandry and Dairying (MoFAH&D), GoI (Facility Registration Number: 77/PO/RcBi/SL/99/CPCSEA). Additionally, the test facility is accredited with a GLP certificate from the National GLP Compliance Monitoring Authority (NGCMA), GoI for the conduct of toxicity studies, and AAALAC International for animal ethics. CPL-II is accredited by National Accreditation Board for Testing and Calibration Laboratories (NABL), GoI, and NGCMA, GoI. The laboratory has an established inhouse quality control program as well as an external quality assessment program with the College of American Pathologists (CAP).

### Compilation of reference intervals and statistical analysis

Reference intervals for hematological and biochemical parameters have been developed according to the C28-A3 guideline for reporting reference intervals of clinical laboratory parameters [24, 25]. Reference intervals have been calculated by detecting outliers, performing normality tests, and employing parametric and non-parametric methods. The distribution of results of the reference population has been estimated at the 2.5th percentile (lower limit) and 97.5th percentile (upper limit). The results of hematology and biochemistry parameters were analyzed using a two-tailed Student’s t-test or Mann–Whitney U test for calculating reference values (GraphPad Prism Software, Version 9.1.1(225)). Statistical analysis was performed to detect significance between individual parameters of inhouse and wild-caught animals and to analyze differences between sexes. Data are presented as mean ± SD. *p*-value less than 0.05 indicated statistical significance.

## Data Availability

All data generated and analyzed is included in this article.

## Abbreviations

AAALAC: American association for accreditation of laboratory animal care
AIDS: Acquired immunodeficiency syndrome
CAP: College of American pathologists
CPCSEA: The committee for the purpose of control and supervision of experiments on animals
CPL-II: Clinical pathology laboratory-II
GLP: Good laboratory practice
GoI: Government of India
HIV: Human immunodeficiency virus
IAEC: Institutional animal ethics committee
K_2_-EDTA: Dipotassium ethylenediaminetetraacetic acid
MoFAH&D: Ministry of fisheries, animal husbandry and dairying
NABL: National accreditation board for testing and calibration laboratories
NGCMA: National GLP compliance monitoring authority
NHP: Nonhuman primates
SD: Standard deviation
SOPs: Standard operating procedures
USA: The United States of America
